# Size-Dependent Thermo- and Photoresponsive Plasmonic Properties of Liquid Crystalline Gold Nanoparticles

**DOI:** 10.3390/ma13040875

**Published:** 2020-02-15

**Authors:** Aleksander Promiński, Ewelina Tomczyk, Mateusz Pawlak, Agnieszka Jędrych, Józef Mieczkowski, Wiktor Lewandowski, Michał Wójcik

**Affiliations:** Laboratory of Organic Nanomaterials and Biomolecules, Faculty of Chemistry, University of Warsaw, Pasteura 1 Street, 02-093 Warsaw, Poland; a.prominski@student.uw.edu.pl (A.P.); etomczyk@chem.uw.edu.pl (E.T.); mr.pawlak@student.uw.edu.pl (M.P.); ajedrych@chem.uw.edu.pl (A.J.); mieczkow@chem.uw.edu.pl (J.M.); wlewandowski@chem.uw.edu.pl (W.L.)

**Keywords:** nanoparticles, liquid crystals, self-assembly, photoresponsive materials, surface plasmon resonance

## Abstract

Achieving remotely controlled, reversibly reconfigurable assemblies of plasmonic nanoparticles is a prerequisite for the development of future photonic technologies. Here, we obtained a series of gold-nanoparticle-based materials which exhibit long-range order, and which are controlled with light or thermal stimuli. The influence of the metallic core size and organic shell composition on the switchability is considered, with emphasis on achieving light-responsive behavior at room temperature and high yield production of nanoparticles. The latter translates to a wide size distribution of metallic cores but does not prevent their assembly into various, switchable 3D and 2D long-range ordered structures. These results provide clear guidelines as to the impact of size, size distribution, and organic shell composition on self-assembly, thus enhancing the smart design process of multi-responsive nanomaterials in a condensed state, hardly attainable by other self-assembly methods which usually require solvents.

## 1. Introduction

In recent years, extensive progress was made in the optimization of nanoparticle (NP) synthetic protocols in order to control their size, shape, and composition. Reaction conditions can now be tuned to yield a specific product with predesigned properties. The development of modern information technology [1,2], energy conversion/transmission [3,4], and light-processing-related applications [5,6] requires advanced materials that meet requirements related to their physicochemical parameters and the control of micro- [7] and nanoscopic organization of structures [8]. For example, electronic, optoelectronic, plasmonic, or fluorescent properties of NPs can be easily controlled by varying their composition and 2D or 3D structure [9,10,11,12,13,14]. Modern lithography techniques (nanoimprint lithography [15] or quantum optical lithography [16]) enable the achievement of materials with sub-10 nm patterns. However, large-scale production of nanoscopic materials using top-down methods and difficulties in obtaining reconfigurable, dynamic self-organization in this way, hamper their use. These problems can be resolved by a bottom-up, self-assembly approach. However, achieving external control over ordered, anisotropic 3D structures using a bottom-up strategy is demanding [17,18].

Throughout the last decade, we put effort into creating nanomaterials based on metal nanoparticles (NPs) modified with functional, liquid-crystal-like organic molecules [19,20,21,22,23,24]. The appropriate molecular architecture of these functional ligands induces a typical liquid-crystalline (LC) organization of NPs [25]. Usually, rod-shaped molecules were studied, but the use of dendrimeric or bent-core molecules was investigated as well [26,27,28]. These types of systems were shown to exhibit various 3D ordered phases that can be controlled with temperature. They possess features characteristic to thermotropic liquid crystals [29,30,31]—softness and anisotropic ordering of the building blocks. The phase sequences of such materials and their structural parameters are strictly dependent on the interplay between the nanoparticle core and the structure of ligands. Several hybrid materials, reconfigurable between ordered lamellar, columnar, cubic, and other types of phases with short- or long-range ordering were obtained [32,33,34,35,36,37].

Unfortunately, thermal control of the dynamic self-assembly of nanoparticles has drawbacks. The main issue is relatively slow switching times, and the need for direct contact of the material with a heat source in order to achieve structural changes. These limitations can be overcome by applying organic ligands which have a photoswitchable structure. Until now, the best results were obtained using photoswitchable azobenzene ligands [38]; e.g., it was possible to achieve dynamic and rapid photoswitchability of liquid-crystal phases using UV light. This approach enabled fast, remote control photoswitchability of phases and recently has been successfully applied to control the structure and plasmonic properties of thin films made of Au 4 nm diameter nanoparticles [39], which seems to be a breakthrough in the context of obtaining dynamic metamaterials.

This promising and convenient approach allows for dynamic and non-contact control over plasmonic information transfer via an optical pathway. Further development of these technologies depends on the optimization of parameters of thin-film systems made of photoswitchable nanoparticles. These parameters depend not only on the structure of organic ligands used to modify the surface of nanomaterials. Other factors—composition of the ligand monolayer, [40] and the presence of an organic matrix—were shown to play a role. The latter approach enabled the achievement of hierarchical structures [41]. One of the key parameters that has to be accounted for, when designing such systems, is the relative size of organic ligands and the nanoparticle core. It is crucial given that the deformation of the nanoparticles’ shape is required for anisotropic self-assembly. In this regard, it should be mentioned, that most of the up-to-date reports on liquid-crystalline phases made of nanoparticles require narrowing the size distribution of nanoparticles (often below 10%) through size-exclusive purification methods [36,39,42,43]. Consequently, the final synthetic yields are relatively low. Further studies of photoswitchability are thus required, particularly in the context of the size and size distribution of nanoparticles. Furthermore, it is essential to achieve systems exhibiting low-temperature photoswitchability, required for future generations of optoelectronic devices.

In the work presented here, we attempted to gain insight into the impact of the ligand structure and the size of nanoparticle cores on the self-assembly process and plasmonic properties of switchable materials. We specifically focused on improving two characteristics important for the applications in plasmon-based devices: (1) lowering the switchability temperature achieved by optimization of the metallic core/organic shell size, and (2) increasing synthetic yields by shortening the workup procedure.

## 2. Materials and Methods

### 2.1. Organic Synthesis of Ligands

All solvents and substrates were purchased from Sigma Aldrich (St. Louis, MO, USA) except for DIAD and 4-chloromethylbenzoyl chloride, which were obtained from TCI. Reagents were used as received, and solvents were dried for 48 h over activated molecular sieves. Synthetic and analytical methods for organic ligands were described before [39].

### 2.2. Small-Angle X-ray Diffraction (SAXRD) Measurements

Small-angle X-ray diffraction (SAXRD) measurements for thin film samples were performed using the Bruker NANOSTAR system (CuKα light source, Bruker, Billerica, MA, USA). Patterns were acquired with the VANTEC2000 area detector placed 68.1 cm from the sample. Samples were drop-casted on Kapton tape. The temperature of the sample was precisely controlled to within 0.1 °C. All materials were heat annealed (by heating to 130 °C and slowly cooling down) before measurements. Temperature-dependent studies were completed in 10 °C temperature steps, using 40 °C/min ramp rate. The signal intensities vs. 2-theta angle were obtained through integration of the pattern over the azimuthal angle using Bruker SAXS software. Diffractograms were fitted using TOPAS3 software. UV irradiation was performed using a Hamamatsu LC8n light source with a fixed intensity of 200 mW/cm^2^.

### 2.3. UV–VIS Spectroscopy

UV–VIS spectra were recorded with a Shimadzu UV-3101 PC spectrophotometer (Shimadzu, Kyoto, Japan), and a LINKAM TP-93 heating stage (LINKAM, Tadworth, UK) was used for temperature control. Samples were deposited on standard microscope cover slides (0.17 mm thick). A Hamamatsu LC8n light source (Hamamatsu, Hamamatsu City, Japan) was used for the UV irradiation.

### 2.4. Thermogravimetric Analysis of Nanoparticles

Thermogravimetric analysis was performed with a TA Q50 V20.13 (TA Instruments, New Castle, DE, USA) analyzer. The measurements were carried out in the temperature range from 20 to 1000 °C with a heating rate of 10 °C /min under a nitrogen atmosphere.

### 2.5. Transmission Electron Microscopy

Transmission electron microscopy (TEM) studies were performed using a Zeiss Libra 120 microscope (Zeiss, Oberkochen, Germany), with a cathode made of LaB6 operating at 120 kV. This device is equipped with OMEGA internal columnar filters. All samples were drop-casted onto 400 mesh carbon film TEM grids, and were heat annealed before measurements.

### 2.6. Synthesis of Gold Nanoparticles

All nanoparticle synthetic protocols were adopted to yield nanoparticles modified with octanethiol for their good solubility in cyclohexane and toluene. Gold nanoparticles with diameter ca. 2 nm were prepared according to the modified Brust–Schiffrin protocol [44] and gold nanoparticles with diameter ca. 5 were prepared according to a modified method reported by Chen and Wang [45]. The synthetic protocols were thoroughly described before [39]. Nanoparticles were purified by an ethanol precipitation method, and finally dissolved in cyclohexane or toluene for 2 nm and 5 nm nanoparticles, respectively. Shape, size, and distribution of obtained nanoparticles were confirmed using TEM studies and SAXRD.

### 2.7. Ligand Exchange Reactions

A ligand exchange reaction was performed at 25 °C using a modified Murray reaction [46]. In order to obtain hybrid materials, ligands were added to 20 mg of octanethiol-coated nanoparticles dispersed in 5 mL of toluene (2 nm NPs) or cyclohexane (5 nm NPs). The NP/L_A_ mass ratio was 1:1 for the 2-L_A_ sample. The NPs/L_M_/L_A_ mass ratio was 1:1:0.5 for the 3-L_M_L_A_ and 5-L_M_L_A_ samples. The reaction was left with gentle stirring at room temperature for 48 hours. During the ligand exchange process there were no signs of nanoparticle aggregation; the solution remained unclouded and formation of precipitate was not observed. Subsequently, nanoparticles were concentrated to 2 mL and sonicated (2 min). Then, the nanoparticles were precipitated with 20 mL of acetone and centrifuged for 10 min at 6000 rpm, and supernatant containing unattached thiol ligands was discarded. The precipitate was dissolved in 1 mL of toluene and centrifuged again. The process was repeated until no traces of unattached ligands remained (determined by thin-layer chromatography, TLC). Finally, samples were redispersed in a small amount of toluene and centrifuged for 10 min at 8000 rpm to remove any aggregates formed during purification. As-prepared NP dispersions were diluted to ~2.5 mg/mL concentration and stored under an inert (nitrogen or argon) atmosphere at 4 °C in the dark. Any given batch of NPs was stored and used for not more than 3 months.

## 3. Results and Discussion

### 3.1. Nanoparticle Design and Preparation

**Figure 1:**
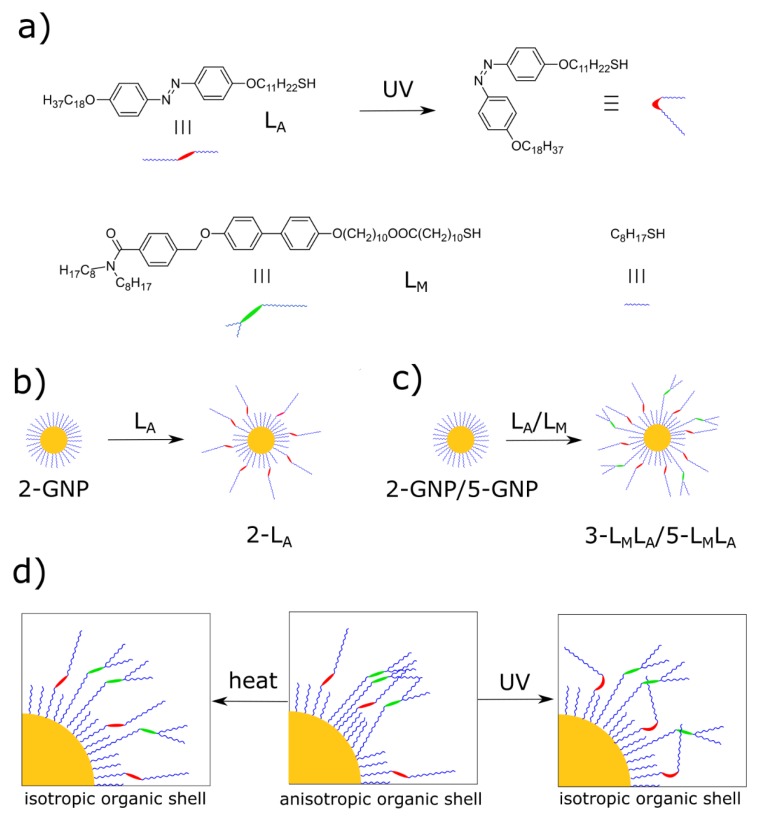
Preparation of photo- and thermo-responsive gold nanoparticle solids: (**a**) structure of a photoswitchable ligand L_A_ and a mesogenic ligand L_M_; (**b**) scheme of introduction of a photoswitchable ligand onto octanethiol-coated 2 nm gold nanoparticles; (**c**) introduction of photoswitchable and mesogenic ligands onto octanethiol-coated 2 nm and 5 nm gold nanoparticles; (**d**) a schematic representation of heat- and UV-induced reorganization of ligands on the surface of gold nanoparticles—the phenomena responsible for reversible reconfiguration of nanoparticle solids.

First, we prepared a set of nanomaterials to investigate the effect of inorganic core size and organic shell composition on reversibly reconfigurable nanoparticle solids. We followed a synthetic strategy which we have recently developed [39]. Shortly, to obtain photoswitchable plasmonic materials, we covered the surface of nanoparticles with stimuli-responsive, liquid-crystalline-like organic ligands, as shown in Figure 1a. We used two ligands that primarily respond to the thermal (L_M_) and optical (L_A_) stimuli. These ligands were synthesized using previously described methods [39].

The architecture of a promesogenic ligand (L_M_) is based on coupling benzyl dioctylamide with a biphenyl group, which results in the formation of a three-ring promesogenic core [29,40]. Our previous research has shown that these ligands, when attached to the surface of nanoparticles, can bundle (schematically shown in the central panel of Figure 1d). This bundling translates to the anisotropic shape of the organic shell and consequently leads to the formation of long-range ordered, anisotropic assemblies of NPs. Moreover, NPs modified with these types of ligands show a strong tendency to induce thermotropic polymorphism of liquid-crystalline phases [29]. Phase transition occurs due to isotropization (melting) of the organic shell of NPs caused by an increase in temperature, as schematically shown in Figure 1d. From the perspective of the current study, a relatively long spacer in the L_M_ molecule was critical. The spacer, which anchors the rigid molecular core to the nanoparticle surface through a flexible hydrocarbon chain, requires an increased adaptability to achieve proper orientation in the assembly process. The overall length of the ligand, in an extended form, is thus ~6 nm, allowing for the formation of ordered assemblies of relatively large nanoparticles (ligands have to be large enough to reorient for anisotropization of the organic shell). Importantly, the ligand is photo- and thermally-stable (for this reason, no aromatic ester linkages were included in its architecture). It is important to note that this ligand does not form liquid-crystalline phases on its own (melts directly to the isotropic phase at 69 °C). However, the architecture of L_M_ is similar to liquid-crystalline compounds, and it supports the formation of LC phases when grafted onto NPs. This effect is attributed to the increased concentration of ligands on the nanoparticle surface and their favorable anisotropic rearrangement [19].

The overall architecture of an azobenzene-based ligand (L_A_) is analogous to the one of L_M_. Namely, L_A_ comprises three segments—the central, biphenyl azobenzene moiety, and two flexible alkyl chains: terminal (endowing the molecule with fluidity) and the linker. The azobenzene moiety can be in the cis and trans states, depending on the illumination conditions, as schematically shown in Figure 1d. Here, we used the shorter linker to attach the azobenzene ligand to the surface of nanoparticles. Thus, the change of the L_A_ geometry is directly affecting the order of ligands in the organic shell. In other words, UV light can change the L_A_ geometry from rod-like (trans configuration) to a bent one (cis configuration), which can sterically disturb L_M_-L_M_ and L_M_-L_A_ bundling phenomena. Details of L_M_ and L_A_ syntheses and characterization of organic ligands are given in the previous contribution.

The sizes of inorganic nanocrystals used for this study were chosen to be smaller and larger than previously reported. Finally, we obtained nanoparticles with ~2 and ~5 nm diameter. The former can be prepared using the Brust–Schiffrin method, and due to their small size should support the formation of 3D ordered assemblies with long correlation lengths [32]. We expect that their small size should also translate to lowering their phase transition temperature [19], possibly enabling achievement of room temperature light-driven reconfigurability. Although these NPs do not exhibit plasmonic properties, we would like to highlight that semiconductor nanocrystals of similar size do exhibit luminescence properties [42], making our proof-of-principle studies interesting for future remote-controlled photonic systems. The latter were synthesized using the dodecylamine-based method. [45] Based on the previous works, we can say that the rule of the thumb is that liquid-crystalline-like ligands can drive the formation of anisotropic assemblies of NPs, the size of which is comparable to the length of the organic molecule [19,33,47,48,49].

In both cases, synthetic methods were adapted to obtain NPs covered with octanethiol (referred to as primary ones). We have chosen to use an alkyl ligand shorter than the usually used dodecanethiol to limit the possibility of hindering the reorientation of stimuli-responsive ligands. Both NP types formed stable colloidal solutions in toluene, even after removal of all unbound ligand molecules. L_M_ and L_A_ ligands were introduced to the surface of NPs via the ligand exchange method. In this process, octanethiol ligands were partially exchanged with the incoming molecules, as shown in Figure 1b,c. The optimized mass ratio for the exchange was 1:1:0.5 Au NPs/L_M_/L_A_ for mixed ligand samples, while a 1:1 mass ratio of Au NPs/L_A_ was used for preparing the sample covered with only one stimuli-responsive ligand—L_A_. All these materials were subject to washing protocols that remove all unbound ligands. We prepared three types of NPs, 2-L_A_, 3-L_M_L_A,_ and 5-L_M_L_A_, in which the number represents the approximated size of the nanocrystal (core size), while L_A_/L_M_ represent types of ligands introduced to the surface (since octanethiol molecules are present in all systems and not included in the names). Details of synthesis of hybrid materials are available in the Materials and Methods section.

Size and polydispersity of nanoparticles of all materials were assessed using transmission electron microscopy (TEM). Analysis shows that all nanoparticles have relatively wide size distribution with polydispersity in the range of 16%–22%. In comparison to the previous works, in which we aimed at size distributions in the order of 10%, the current work enables us to explore a more industrially relevant approach, in which the final material does not undergo a thorough purification (precipitation–centrifugation–redispersion) procedure. The crucial benefit is limiting gold loss in the synthetic process. It is worth noting that the size of NPs changed in the course of the ligand exchange reaction. This phenomenon can be explained on the grounds of a ripening/coalescence process of NPs driven by the excess thiols [50]. As a result, 2.2 nm primary NPs after modification with L_A_ yield hybrid material 2-L_A_ with a slightly larger diameter of 2.3 nm. Modification with a mixture of L_A_ and L_M_ led to nanoparticles with a diameter of 2.9 nm (3-L_M_L_A_). A significant increase in diameter is caused by a larger thiol concentration and longer exchange time. In the case of nanoparticles with a starting diameter of 5.0 nm, no change in the core diameter was observed after the ligand exchange reaction. Increased stability of this sample to thiol-induced ripening is due to the lower surface energy of NPs.

In addition to TEM, NPs were characterized using thermogravimetric analysis (TGA) and UV–VIS spectroscopy to confirm the composition, thermal stability, and plasmonic properties of the materials. The results of these measurements are summarized in Table 1. TGA thermograms showed that in all cases, organic content was above 34%, which is high in comparison to values achieved for alkyl coated NPs [27,39], thus confirming the successful incorporation of relatively large organic ligands onto the NPs’ surface. It is worth noting that the larger the NPs, the lower the relative amount of the organic part, which is reasonable given that the mass of the metallic core increases with the third power of the radius, while the amount of the attached ligands changes with the second power (the change of surface area). Additional factors, such as curvature-dependent ligand density, affect the exact results, however not as significantly as the radius size alone. Similar to the previously analyzed materials, the observed weight losses could be attributed to three temperature regions and corresponding processes: 200 and 250 °C when octanethiol molecules are removed, between 250 and 320 °C when it can be observed that azobenzene derivative L_A_ decomposes, and finally above 320 °C when the promesogenic ligand L_M_ decomposes.

### 3.2. Thermally Driven Self-assembly of Nanoparticles

To investigate the self-assembly of NPs, we used small-angle X-ray diffraction (SAXRD). Importantly, the set up was designed to investigate NP organization changes in situ under thermal or light stimuli. All analyses were performed on solid-state samples of NPs drop-casted onto Kapton film from toluene dispersions. Samples were first subject to thermal annealing (heating to 130 °C and cooling down to room temperature at 3 °/min rate) to obtain large well-ordered domains (in other words, to achieve long correlation lengths of NP positions). SAXRD measurements were carried out in the function of temperature and light irradiation.

**Figure 2:**
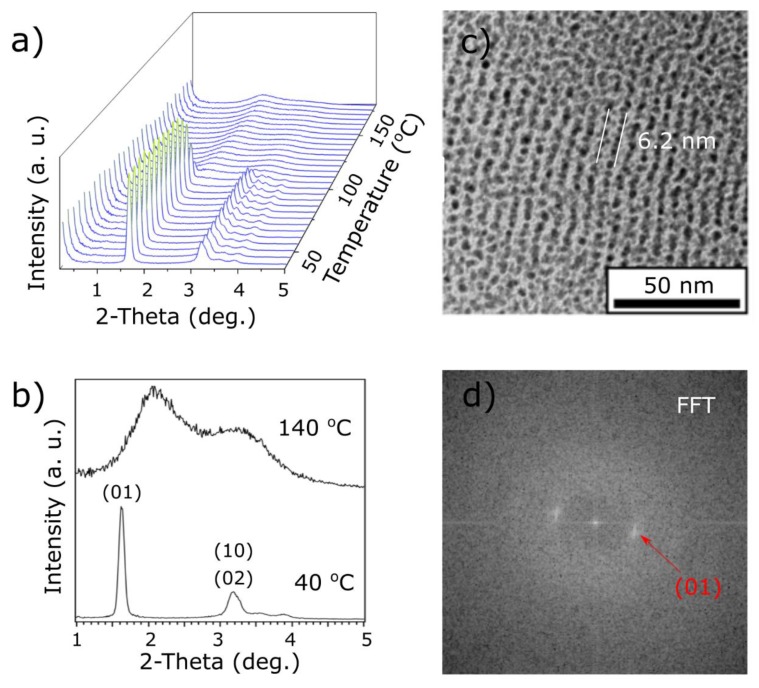
Small-angle X-ray diffraction (SAXRD) and TEM analysis of 2-L_A_ material. (**a**) Temperature evolution of 1D SAXRD profile during heating. (**b**) Comparison of 1D XRD diffractograms collected at 40 and 140 °C. (**c**) TEM image of a heat annealed material. (**d**) 2D Fast Fourier Transformation (FFT) of image shown in (**c**) with maximum assigned to (01) (red arrow).

For small nanoparticles with 2.3 nm diameter, covered with the azobenzene ligand (2-L_A_), relatively strong and narrow X-ray diffraction (XRD) signals were observed at room temperature, as shown in Figure 2b, at 5.3 and 2.8 nm periodicities. The obtained structure can be fitted with low error to a lamellar-type configuration of NPs with interlayer distance 5.3 nm (based on the position of the main XRD peak assigned as (01)). The in-layer spacing (10) signal is observed at ~2.8 nm (overlaying with the (02) peak). These periodicities can be translated to the NP surface-to-surface distances—3.0 and 0.5 nm, in directions along and perpendicular to the layer normal. Obtained values are expected when interpenetration of organic shells of neighboring NPs is taken into account and suggests that the in-layer distance is determined by the alkyl thiols, while interlayer by larger, L_A_ ligands.

Under heating, the observed diffractogram changed, as shown in Figure 2a. Below 100 °C, a small shift of the main signal (01) was observed. It is evidence of the shortening of the interlayer distance and suggests an ongoing reorientation of the ligands in this process. Above 100 °C, the diffractogram changed distinctly. We observed two relatively wide XRD signals, as shown in Figure 2b, in positions that can be assigned to the short-range hexagonal arrangement, leading to a conclusion that the organic shell of the NPs adopts a spherical shape in this phase and a close-packed type arrangement of NPs forms. The average interparticle distance in the hexagonal phase is approximately 5.0 nm. It is shorter than the interlayer distance of the lamellar phase because, at this temperature, the ligands are no longer bundled. Above 180 °C, a slow decomposition and aggregation of nanoparticles, which can be attributed to Ostwald ripening of NPs, is characterized by a strong scattering signal near the beam-stop and complete disappearance of diffraction peaks.

To further confirm the formation of anisotropic assemblies by 2-L_A_ NPs, we used TEM imaging. In analogy to XRD measurements, samples were first drop-casted onto a TEM grid (coated with a carbon film) and then heat annealed. In some parts of the TEM grid, we observed structures that could be assigned to layers of NPs arranged in a way that the layer normal is in the plane of observation of the TEM apparatus of NPs, as shown in Figure 2c, confirming that 2-L_A_ NPs tend to arrange into anisotropic structures. Quality of order was further confirmed by observing sharp, discrete signals after 2D Fast Fourier Transformation of the TEM image, as shown in Figure 2d, corresponding to the (01) XRD signal. The interlayer distance measured in the ordered areas was ~6.2 nm, which is larger than the XRD derived value. However, given the relatively wide dispersity of NP sizes and the NPs’ tendency to size-segregate, the difference comes from imaging of only a subset of larger NPs. Another explanation is that only small domains were oriented as described above, which can translate to local deformation of the favored structure. Yet another possibility is that the phase transition is not fully reversible, and thus we observe the effect of partial, kinetic trapping of the upper temperature phase, or the observations reflect subtle differences in the heat annealing process of SAXRD and TEM samples (regarding different appliances used for heat annealing and substrates with different thermal conductivity). Nevertheless, TEM imaging indicates the tendency of 2-L_A_ NPs to form anisotropic arrangements, thus supporting the XRD results.

**Figure 3:**
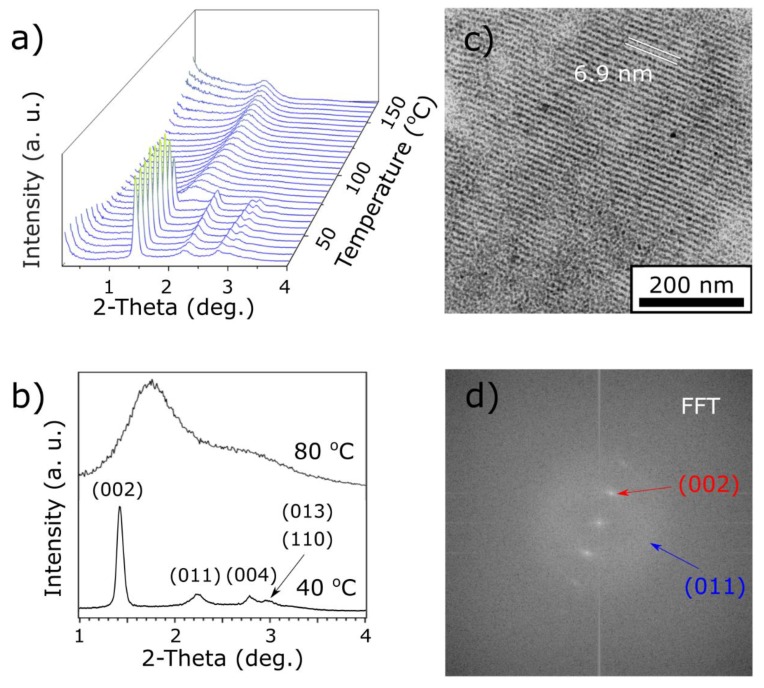
SAXRD and TEM analysis of 3-L_M_ L_A_ material. (**a**) Temperature evolution of the 1D SAXRD profile during heating. (**b**) Comparison of 1D XRD diffractograms collected at 40 and 80 °C. (**c**) TEM image of the heat annealed material. (**d**) 2D Fast Fourier Transformation of the image shown in (c) with maxima assigned to (002) (red arrow) and (011) (blue arrow) lattice planes.

SAXRD measurements of a heat annealed 3-L_M_L_A_ sample, as shown in Figure 3a, revealed that it also self-assembled into anisotropic, long-range ordered assemblies. At 30 °C, four distinct XRD peaks were observed at periodicities of 6.4, 4.0, 3.2, and 3.0 nm. These were indexed as (002), (011), (004), and a pair of (013) and (110) signals, assuming a tetragonal body-centered unit cell with unit cell parameters c ≈ 12.8 nm and a ≈ 4.2 nm. These observations were in agreement with the already shown tendency of liquid-crystalline-like ligand coated NPs to adopt this symmetry [27,36,42] as well as interparticle spacing. Surface-to-surface distance of neighbor NPs is ~0.9 nm along the *a* direction, and 3.7 nm, along the unit cell diagonal direction. In comparison to the 2-L_A_ sample, these distances are larger, which is expected given the larger molecular size of L_M_ ligands compared to L_A_ molecules.

When the sample was heated, the observed signals changed at 70 °C. Interestingly, the sample coated with mixed ligands had significantly lower transition temperature than 2-L_A_, which we attribute to the lower melting point of L_M_ compound (69 °C) in comparison to L_A_ (105 °C), as well as steric incompatibility of L_A_ and L_M_ ligands at the surface of NPs, translating to a more fluidic nature of the organic shell. In other words, L_A_ ligands can efficiently form bundles on the surface of L_A_ NPs, which requires more energy to melt than a less ordered mixed ligand shell. At 80 °C, the diffractogram showed two relatively broad XRD signals at position 5.4 and 3.1 nm, as shown in Figure 3b, which were indexed as (01) and (11) signals of a 2D, short-range ordered, hexagonal packing of spheres with a ≈ 6.2 nm. This arrangement requires the surface-to-surface distance of ~3.1 nm, which suggests a reorientation of ligands around the central core. Heating above 130 °C caused increased scattering around the beam-stop, due to aggregation of nanoparticles, which finally rapidly decomposed above 175 °C.

TEM measurements revealed the presence of large (up to 600 nm diameter) domains of NPs, as shown in Figure 3c. These assemblies had a strong tendency to form layers of NPs arranged in a way that the layer normal is in the plane of observation of the TEM apparatus with interlayer distance of ~6.9 nm, corresponding well to the (002) periodicity calculated from the XRD measurements (~6.4 nm). The long-range ordered nature of the sample is shown in the Fast Fourier Transformation (FFT) analysis of the TEM image, as shown in Figure 3d, which revealed a series of discrete signals corresponding to the interlayer spacing. Notably, also weakly developed diagonal signals are observed, which correspond to (011) XRD signals, confirming the correlation of positions of NPs in consecutive layers. We note that consistency of TEM and SAXRD results suggests that the observed phase transitions are fully reversible.

**Figure 4:**
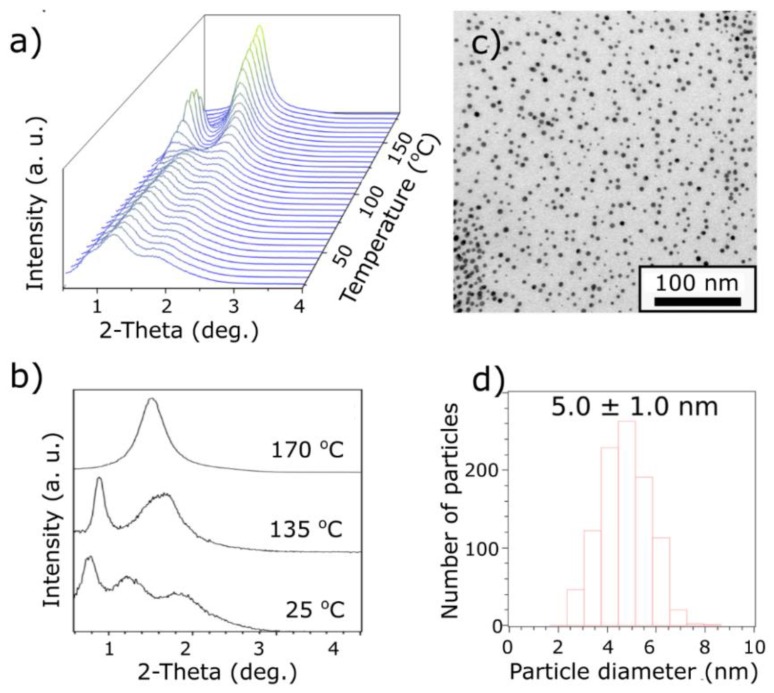
SAXRD and TEM analysis of 5-L_M_L_A_ material. (**a**) Temperature evolution of SAXRD peaks during heating; (**b**) comparison of XRD peak location at temperatures of 25°C, 135°C, and 170°C as obtained by integration of suitable patterns; (**c**) TEM image of material; (**d**) histogram of size distribution of 5-L_M_L_A_ nanoparticles

Finally, we tested the 5-L_A_L_M_ sample using XRD analysis. At 30 °C, we observed a series of three, relatively broad XRD signals at 11.0, 7.2, and 4.8 nm, as shown in Figure 4a,b. This pattern is fitted to the model in which NPs form a body-centered tetragonal phase with unit cell parameters of c ≈ 22.0 and a ≈ 7.4 nm. In this case, the experimentally observed signals were indexed as (002), (011), and (112). It is important to note that since the XRD signals are relatively broad, there exists a possibility of another fitting solution. To exclude false-positive results, we considered structures usually formed by liquid-crystal-like ligand coated NPs, e.g., 3D ordered close-packed, tetragonal, orthorhombic, and hexagonal close-packed phases [36,48,49]. However, none of these structures produced a satisfactory fit with acceptable dimensions of the unit cells. The existence of the tetragonal phase is further supported by the fact that 3 nm NPs, with the same organic shell, adopt similar symmetry. The heating of the sample results in the deformation of the unit cell. At 110 °C, the unit cell parameters are c ≈ 19.0 and a ≈ 8.1 nm. Under further heating, NPs reorganize and exhibit a lamellar phase in the range of 120 to 150 °C with 9.9 nm interlayer and 6.4 nm in-layer spacings, as shown in Figure 4a,b. These periodicities correspond to the NP surface-to-surface distances of 4.9 and 1.4 nm in directions along and perpendicular to the layers normal. Above 150 °C, a single, broad XRD peak is observed with 5.8 nm periodicity, indicating the existence of an isotropic phase, as shown in Figure 4a,b.

TEM analysis of the 5-L_A_L_M_ sample confirmed that these NPs exhibit broad size distribution (20%), as shown in Figure 4d, of Gaussian shape. TEM image analysis of the heat annealed sample, as shown in Figure 4c, did not reveal the formation of a long-range ordered structure as in the case of the 2-L_A_ and 3-L_A_L_M_ samples. Instead, short 1D chains of NPs were observed, which could be the result of NPs’ tendency to assemble or an artifact of evaporation. Even in the latter case, it is worth analyzing interparticle spacing, since it reflects the shape of the organic shell after the heat annealing procedure. The lowest interparticle distances in such arrangements are ~6 nm, corresponding well to the interparticle distance measured in the lamellar phase, and most of these distances are found in the 6–10 nm range, similar to interparticle spacing in various phases as derived from XRD studies. Such variability of the distances is the effect of short-range order character of NP arrangements, which translates to a non-equilibrium deformation of the organic shell, and also stems from the wide size distribution of NPs. Therefore, our TEM measurements deviate from the mean values determined by XRD. Still, the tendency of 5-L_A_L_M_ NPs to form 1D chains and the values of interparticle distances corresponding to XRD measurements indicate anisotropization of the organic shell due to liquid-crystal-like ligands bundling.

It is relevant to note the different self-assembly modes of NPs covered with the L_A_L_M_ monolayer. In the case of 3, 4 (reported in ref. [39]), and 5 nm diameter Au nanocrystal cores, various phases and phase transition temperatures are observed. The overall tendency is that the larger the nanocrystal core, the higher temperatures are required to observe reconfiguration of anisotropic aggregates of NPs into short-range or close-packed structures, in analogy to some of the previously reported LC NP systems [43,50]. These observations are consistent with the fact that increasing the size of nanocrystals translates to a larger volume fraction of Au; this, in turn, increases van der Waals interactions between nanocrystals and density of the material.

### 3.3. Light-driven Self-assembly of Nanoparticles

**Figure 5:**
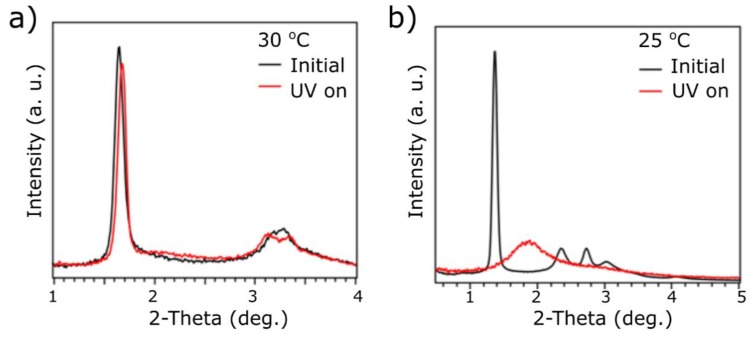
SAXRD photoswitching measurements of (**a**) 2-L_A_ and (**b**) 3-L_M_L_A_ samples.

All three materials were subjected to in situ SAXRD studies under UV irradiation. Previously, it has been shown that samples without light-responsive (i.e., azobenzene derivative) ligands do not exhibit a change in the position and intensity of X-ray signals. In our study, all NPs contain photoresponsive ligands in their shell, but only one is unsupported by the mesogenic ligands. In the case of the smallest nanoparticles, 2-L_A_, UV-light irradiation induces small structural changes. We observed a small change of the position of the main peak (01) by 0.6 nm, indicating a reduction of the interlayer spacing between particles, while the in-layer spacing was not affected, as shown in Figure 5a. At the same time, the shift of the (02) signal enables clear discrimination of position from the otherwise overlayed (10) XRD signal, thus further supporting the lamellar phase assignment.

In the case of 3-L_M_L_A_ nanoparticles, as shown in Figure 5b, a drastic change of the diffractogram was observed when the sample was irradiated with UV light. Under irradiation, the diffractogram comprised two, relatively broad XRD signals positioned at 4.8 and 2.8 nm, which could be indexed as (01) and (11) signals coming from a 2D, short-range ordered, hexagonal packing of spheres with the mean interparticle distance ~5.5 nm. Notably, in comparison to the 2D hexagonal phase formed at 80 °C, the interparticle spacing of the irradiated assembly is reduced. We assign this effect to the two different mechanisms of organic shell isotropization in those samples. Heating causes the melting of both ligands, leading to higher steric demands per molecule [36], while UV light irradiation disturbs the packing of the molecules by changing the symmetry of the L_A_ ligand, but most probably do not increase their steric demands.

Irradiation of the 5-L_M_L_A_ sample did not induce changes of the diffractogram, indicating that in this case, the change of the geometry of azobenzene ligands is not sufficient to induce isotropization of the organic shell.

### 3.4. Switchable Plasmonic Properties of Nanoparticles

**Figure 6:**
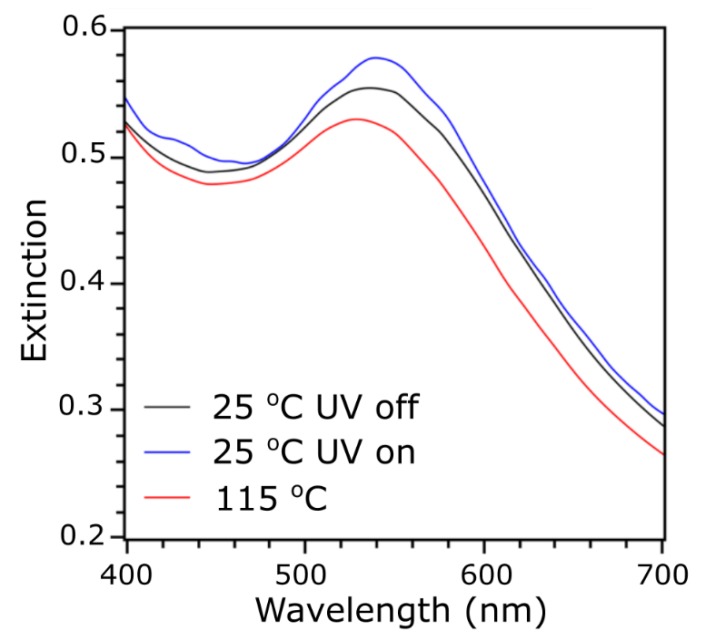
UV–VIS analysis of 5-L_M_L_A_ material thermo- and photo- dependent plasmonic properties.

Finally, we aimed at probing the plasmonic properties of nanomaterials under UV light irradiation. The 2-L_A_ and 3-L_M_L_A_ samples do not exhibit significant localized surface plasmon resonance (LSPR), so only the 5-L_M_L_A_ material was studied, as shown in Figure 6. The material was drop-casted onto a glass slide, which was mounted onto a Peltier-type heating stage and heat annealed similarly to the XRD samples. A UV–VIS detector was positioned orthogonally to the incoming UV light beam. Measurements of the sample exhibiting the tetragonal phase show a plasmonic band with an absorption maximum at ~540 nm. Redshift of the band, compared to the ~520 nm maximum for the dispersions of these NPs, indicates the coupling of the plasmons in 5-L_M_L_A_ nanoparticle solid. After heating the sample to 125 °C, an 8 nm blueshift of the plasmonic band maxima was observed. The result is consistent with the growth of nearest neighbor distance between NPs with changing parameters of the tetragonal phase unit cell (i.e., increase of the *a* parameter) [36,39,51]. As expected from XRD results, there was almost no shift observed for nanoparticles at 25 °C after UV irradiation, as no clear structural changes occur under these conditions. Based on our previous work, we know that if a sample of liquid-crystal-like ligand coated nanoparticles exhibits reversible switching of the structure, the reversible switching of the plasmonic band maxima is also observed [39,43,51].

## 4. Conclusions

In this study, we have prepared a series of nanoparticles coated with liquid-crystal-like ligands comprising UV-light responsive moieties. The Au metallic core size varied from 2.3 to 5.0 nm. All samples exhibited a high dispersity of size, which was necessary to obtain high yield production of these materials. Nonetheless, we observed the formation of various 2D and 3D long-range ordered structures, evidencing that the adopted low-cost approach to functional nanomaterials is feasible. Importantly, all samples exhibit thermal switchability; between lamellar and short-range hexagonal, tetragonal and short-range hexagonal, tetragonal and lamellar phases for samples 2-L_A_, 3-L_A_L_M,_ and 5-L_A_L_M_, respectively. One of the samples, equipped with L_A_ and L_M_ ligands and 3.1 nm core size, also exhibits clear light-responsive structural properties at room temperature. Specifically, it can be rearranged between 3D body-centered tetragonal and short-range hexagonal phases by UV light irradiation. Importantly, the UV light-dependent behavior occurred already at 25 °C, which makes this responsiveness of particular importance in device applications. Although this sample does not exhibit plasmonic properties, these findings are relevant for obtaining semiconductor nanocrystal solids with remotely controlled structure and emission properties [42]. Additionally, this work shows that the recently proposed [39] organic shell composition does not allow low temperature, light-driven reorganization of ~5 nm diameter NPs, setting the size threshold for currently explored organic mixtures. Our findings suggest that further studies for the development of more fluid ligands, e.g., by the incorporation of branched alkyl chains into their structure, is required to access materials based on NPs larger than 5 nm in diameter. Finally, we believe that our findings outline general design rules for achieving relatively low cost, light- and thermo-reconfigurable nanoparticle solids working at room temperature. Namely, thermal switchability can be achieved for samples in which NPs with size distribution in the order of 15%–20% if ligands are of the size comparable to the NPs. However, achieving low-temperature photo-induced reconfiguration is not as easy—it can be achieved for NPs smaller than the length of elongated ligands. We envision that the introduction of more fluid terminal chains (e.g., oleyl chains) or managing the length of the azo-comprising compounds is required to shift the size threshold above which low-temperature, UV-irradiation susceptibility is achieved.

## Figures and Tables

**Table 1:** Size, composition, and optical properties of nanoparticles (NPs). LSPR—localized surface plasmon resonance, ND—not determined, *—at 30 °C in toluene dispersion.

Sample	Core size (nm) from TEM	Mass % of Organics from TGA	LSPR (nm)*
2-L_A_	2.3 ± 0.4	~47.2	ND
3-L_A_L_M_	3.1 ± 0.5	~43.7	ND
5-L_A_L_M_	5.0 ± 1.1	~34.8	520
